# Evolutionary phylodynamics of foot-and-mouth disease virus serotypes O and A circulating in Vietnam

**DOI:** 10.1186/s12917-016-0896-0

**Published:** 2016-11-29

**Authors:** Van Phan Le, Thi Thu Hang Vu, Hong-Quan Duong, Van Thai Than, Daesub Song

**Affiliations:** 1 Department of Microbiology and Infectious Disease, Faculty of Veterinary Medicine, Vietnam National University of Agriculture, Hanoi, Vietnam; 2 Research and Development Laboratory, Rural Technology Development JSC, Hung Yen, Vietnam; 3 Institute of Research and Development, Duy Tan University, Danang, Vietnam; 4 Department of Microbiology, Chung-Ang University College of Medicine, Seoul, South Korea; 5 College of Pharmacy, Korea University, Sejong, South Korea

**Keywords:** Foot-and-mouth disease virus, Serotype O, Serotype A, Vietnam

## Abstract

**Background:**

Foot-and-mouth disease virus (FMDV) is one of the highest risk factors that affects the animal industry of the country. The virus causes production loss and high ratio mortality in young cloven-hoofed animals in Vietnam. The VP1 coding gene of 80 FMDV samples (66 samples of the serotype O and 14 samples of the serotype A) collected from endemic outbreaks during 2006–2014 were analyzed to investigate their phylogeny and genetic relationship with other available FMDVs globally.

**Results:**

Phylogenetic analysis indicated that the serotype O strains were clustered into two distinct viral topotypes (the SEA and ME-SA), while the serotype A strains were all clustered into the genotype IX. Among the study strains, the amino acid sequence identities were shared at a level of 90.1–100, 92.9–100, and 92.8–100% for the topotypes SEA, ME-SA, and genotype IX, respectively. Substitutions leading to changes in the amino acid sequence, which are critical for the VP1 antigenic sites were also identified. Our results showed that the studied strains are most closely related to the recent FMDV isolates from Southeast Asian countries (Myanmar, Thailand, Cambodia, Malaysia, and Laos), but are distinct from the earlier FMDV isolates within the genotypes.

**Conclusions:**

This study provides important evidence of recent movement of FMDVs serotype O and A into Vietnam within the last decade and their genetic accumulation to be closely related to strains causing FMD in surrounding countries.

**Electronic supplementary material:**

The online version of this article (doi:10.1186/s12917-016-0896-0) contains supplementary material, which is available to authorized users.

## Background

Foot-and-mouth disease virus (FMDV), causing foot-and-mouth disease (FMD), is a contagious virus affecting cloven-hoofed domestic (pig, cattle, goat, and sheep) and wild animals. FMDV has been detected in >100 countries worldwide, mostly in Asia, Africa, and the Middle East [1]. The FMD causes economic losses to the livestock population and reduces food security and economic development. For this reason, FAO and OIE have launched a necessary strategy for global FMD control.

FMDV, a picornavirus, is the prototypical member of the *Aphthovirus* genus within the *Picornaviridae* family. The virus particle is about 25–30 nm in diameter and roughly spherical in shape [2]. Similar to that of other picornaviruses, the FMDV genome organization consists of a large single open reading frame that encodes for the structure proteins, VP4, VP2, VP3, and VP1 (also known as 1A, 1B, 1C, and 1D, respectively), in which VP1, VP2, and VP3 are surface proteins; while VP4 is located internally.

FMDV is well identified as having seven immunological distinct serotypes, including serotype O, A, C, Asia 1, and the South African Territories (SAT) serotypes (including SAT1, SAT2, and SAT3) subsequently with numerous identified subtypes [3]. The infection of a single viral serotype does not confer, in consequence, the full protection against the infection of other viral serotypes [4]. The FMDV serotype A has been considered to be one of the most antigenically diverse among the seven serotypes [5, 6]. The FMDV serotype A has been classified into 10 major genotypes (designated as I to X) based on the VP1 phylogenetic trees [5, 6]. FMDV serotype O is classified into 11 topotypes, designated as Europe-South America (Euro-SA), Middle East-South Asia (ME-SA), Southeast Asia (SEA), Cathay (CHY), West Africa (WA), East Africa 1 (EA-1), East Africa 2 (EA-2), East Africa 3 (EA-3), East Africa 4 (EA-4), Indonesia-1 (ISA-1), and Indonesia-2 (ISA-2) [7, 8]; and FMDV serotype Asia 1 is classified into seven genotypes (designated as I to VII) [9].

FMD is an endemics and widespread disease in African, Asia, and its further spread into the FMD-free areas like American, Europe, and Australia is a direct threat [1]. Among seven serotypes of FMDV, serotype O and A have been distributed extensively and are responsible for outbreaks in Asia and Africa; the three SAT serotypes have been generally restricted in their distribution to Africa, and the serotype Asia 1 has never been found outside of Asia [10, 11].

Agriculture plays an important role in the national economy of Vietnam, in which animal production contributes about approximately 32% to the total GDP. FMD is considered the most economically important infectious disease affecting domestic cattle, buffalo, and pigs. The concurrent circulation of FMDV serotypes O, A, and Asia 1 are detected in which the serotype O remains the most prevalent and is responsible for the highest numbers of outbreaks [12, 13]. The FMDV serotype O and its outbreak in Vietnam were first described in academic research between 1996 and 2001 [14]. The FMDV serotype Asia 1 and A were subsequently identified in 2005 and 2009, respectively [12, 15]. In 2008 and the first 2 months of 2009, the FMDV serotypes O and A were reported to be the prevalent serotype and caused approximately 166 FMD outbreaks in 128 communes in 47 districts of 14 provinces throughout the country [16].

Currently, limited information is available regarding the genetic characteristics and geographical distribution of the FMDV serotype O and A causing sporadic outbreaks in Vietnam. In this study, the VP1 coding gene of 80 FMDV samples (66 samples of the serotype O and 14 samples of the serotype A) collected from endemic outbreaks during 2006–2014 were analyzed to investigate their phylogeny and genetic relationship with other available FMDVs globally. These data will provide important evidence of recent movement of FMDVs serotype O and A into Vietnam within the last decade and their genetic characterization with strains causing FMD in neighboring countries.

## Methods

### Sample collection and virus isolation

**Fig. 1:**
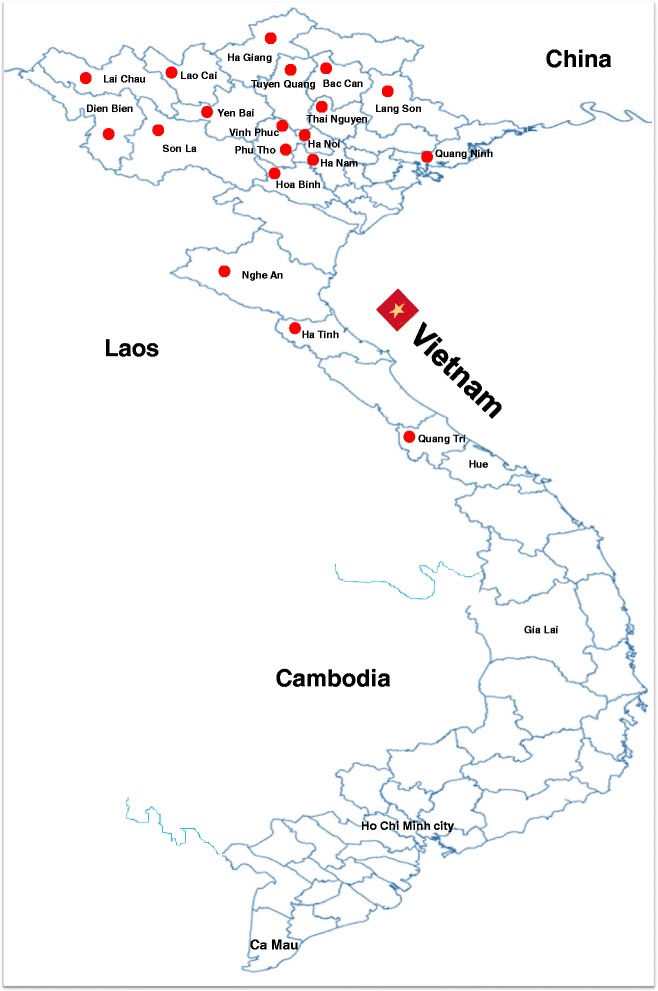
Map of Vietnam showing the provinces (*red*) from which the FMDV isolates were collected during outbreaks

A total of 80 FMD-positive samples were collected in a passive surveillance program from 19 provinces located in north and northern central Vietnam during 2006–2014 (Table 1, Fig. 1). All the virus isolates were initially confirmed by FMDV antigen ELISA (WRL Pirbright, UK) and then isolated from the BHK-21 cell culture system with subsequence passages. Briefly, BHK-21 cells were cultured in minimum essential medium (MEM; Gibco BRL, Grand Island, NY, USA) supplemented with 5% fetal bovine serum (FBS; Gibco BRL) and 0.1% Gentamicin (Gentamicin Reagent Solution, Gibco BRL) at 37 °C in a humidified atmosphere containing 5% CO_2_. The epithelial homogenate was centrifuged at 10,000 g for 10 min, and the supernatant was then filtered by using a 0.45-μm sterile syringe filter (Corning Costar, Corning, NY, USA). The filtered samples were inoculated into a monolayer of BHK-21 cells at 37 °C for 1 h, followed by two washes with MEM media. The infected cells were maintained in MEM media containing 2% FBS. Infected cells were harvested after 2–4 days post-infection and were subsequently passed into the BHK-21 cells until cytopathic effects appeared. The names of the isolates were assigned as follow: serotype, country code, laboratory record number, and the year of sample collection, and stored at −80 °C for further exam (Additional file 1).

**Table 1:** Origin of the serotypes O and A FMDVs sisolated in this study

Year ofisolation	Province	Host	Number of sample	Type	Topotype
2006	Vinh Phuc	Buffalo	1	O	SEA
	Thai Nguyen	Buffalo	1	O	SEA
		Cattle	2	O	SEA
	Ha Noi	Cattle	1	O	SEA
		Pig	1	O	SEA
	Lang Son	Cattle	1	O	SEA
		Pig	1	O	SEA
	Son La	Cattle	5	O	SEA
		Pig	1	O	SEA
		Buffalo	2	O	SEA
2007	Lai Chau	Buffalo	1	O	SEA
	Thai Nguyen	Cattle	2	O	SEA
	Ha Tinh	Cattle	1	O	SEA
2008	Nghe An	Buffalo	3	A	Genotype IX
		Cattle	1	A	Genotype IX
2009	Yen Bai	Buffalo	2	O	SEA
	Son La	Buffalo	6	O	SEA
		Pig	1	O	SEA
	Ha Giang	Cattle	1	O	SEA
	Tuyen Quang	Buffalo	1	O	SEA
	Nghe An	Cattle	1	O	SEA
	Quang Ninh	Buffalo	2	O	SEA
	Hoa Binh	Buffalo	3	O	SEA
		Cattle	2	O	SEA
		Buffalo	1	A	Genotype IX
	Lang Son	Buffalo	1	O	SEA
	Lao Cai	Cattle	1	O	SEA
	Phu Tho	Cattle	2	O	SEA
		Buffalo	1	A	Genotype IX
	Bac Can	Buffalo	1	A	Genotype IX
		Cattle	1	A	Genotype IX
	Ha Giang	Cattle	2	A	Genotype IX
	Quang Tri	Buffalo	1	A	Genotype IX
2010	Son La	Buffalo	2	O	SEA
	Lao Cai	Buffalo	1	O	SEA
	Dien Bien	Cattle	1	O	SEA
	Tuyen Quang	Buffalo	1	O	SEA
	Yen Bai	Cattle	2	O	SEA
		Buffalo	1	O	SEA
2013	Ha Noi	Pig	1	O	ME-SA
		Bovine	2	A	Genotype IX
	Quang Tri	Bovine	4	O	ME-SA
	Ha Tinh	Bovine	1	A	Genotype IX
2014	Ha Noi	Pig	6	O	ME-SA
	Ha Nam	Pig	3	O	SEA
		Bovine	1	O	SEA

### RNA extraction and reverse transcription-polymerase chain reaction (RT-PCR)

The viral RNA was extracted from infected cell culture supernatants using the QIAamp viral RNA mini kit (Qiagen, Valencia, CA, USA), according to the manufacturer’s instructions. The cDNA step was performed using the Superscript™ III First-Strand Synthesis System for RT-PCR (Invitrogen, Carlsbad, CA, USA) according to the manufacturer’s instructions. The primer set of the VN-VP1F/VN-VP1R (VN-VP1F: 5′-AGYGCYGGYAARGAYTTTGA-3′, VP1R: 5′-CATGTCYTCYTGCATCTGGTT-3′) was used for the PCR-amplification of DNA fragments containing the 639 nt length of the VP1 coding region [17]. Briefly, the reaction was carried out at 42 °C for 60 min (reverse transcription), 35 cycles of 95 °C for 1 min (for denaturation), 52 °C for 1 min (for annealing), and 72 °C for 1 min (for extension), followed by 72 °C for 10 min (for final extension). The PCR products were separated on 1.2% SeaKem LE agarose gel and viewed on a BioRad Gel Doc XR image-analysis system.

### Nucleotide sequencing and sequence analysis

Capsid VP1 is the most studied FMDV protein because of its significance for virus attachment and entry, protective immunity, and serotype specificity [3, 18, 19]. The amplified capsid VP1 PCR products were either purified with QIAquick PCR purification kit or QIAquick gel extraction kit according to the manufacturer’s instructions (Qiagen). RT-PCR primers were used for the direct sequencing of internal gene segments by using a BigDye terminator cycle sequencing kit and an automatic DNA sequencer (Model 3730, Applied Biosystems, Foster City, CA, USA). The obtained nucleotide and deduced amino acid sequences in this study were aligned using the ClustalX 2.1 program [20] and Lasergene software (DNASTAR; Madison, WI, USA) by using the parameters set against the corresponding FMD viral sequences from the NCBI GenBank.

### Phylogenetic analysis

The complete nucleotide sequences of the VP1 coding gene from the 80 FMDV samples examined in this study were compared against a representative VP1 coding gene from the available FMDV sequences in the GenBank database. Phylogenetic trees were constructed by the neighbor-joining algorithm put into practiced with MEGA 6.06 software suite [21]. The bootstrap resampling method with 1000 replicates was used to evaluate the topology of the phylogenetic tree. The pairwise distance was calculated using MEGA 6.06 software package [21].

## Results

### Detection of FMDV genome and genome sequencing

The viral RNA was sufficiently extracted from 80 FMDV samples (66 samples of the serotype O and 14 samples of the serotype A) (Table 1). The VP1 coding gene was successfully amplified by RT-PCR using the primer set as described above. The full length sequence of the VP1 coding gene was determined by direct sequencing of the PCR amplicons. Thereafter, the obtained nucleotide sequences have been deposited in the NCBI GenBank database under the accession numbers described in Additional file 1.

### Genetic diversity and phylogenetic analyses of the VP1 coding gene

**Fig. 2:**
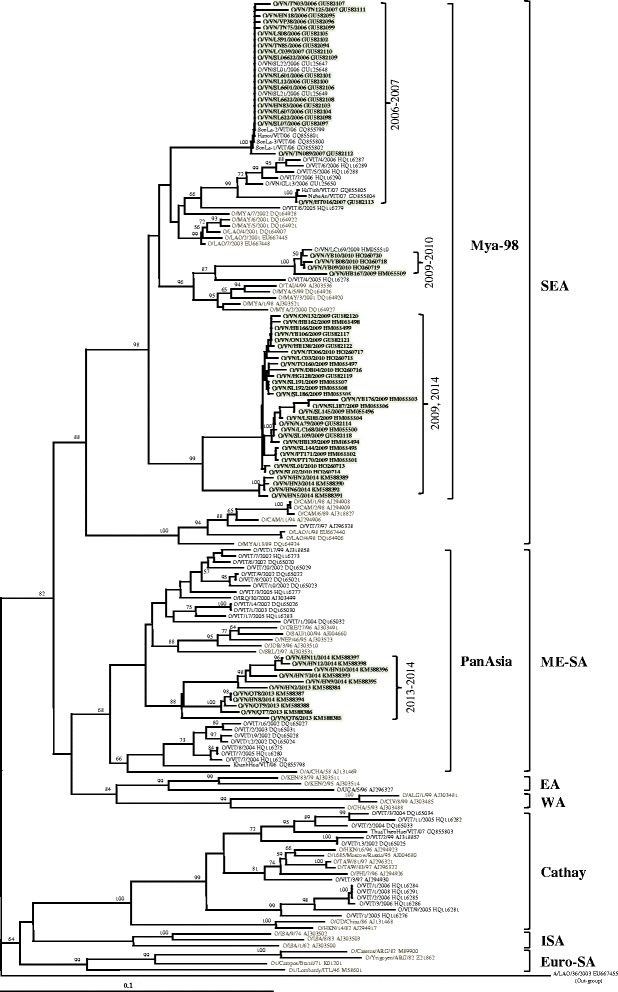
Phylogenetic tree based on the complete nucleotide sequence of the VP1 coding region of type O FMDVs showing relationships between the study strains and other type O representatives worldwide. The Vietnamese strains are marked in *bold*. *Numbers* at nodes indicate the level of bootstrap support based on the neighbor-joining analysis of 1000 resampled datasets. Only values above 50% are given. A *bar* represents 0.1 substitutions per nucleotide position

For the FMDV serotype O, the nucleotide sequence identity among 66 FMDV serotype O isolates showed diversity at a level of 79.1–100%. These strains shared nucleotide sequence identity at 100, 91.5–97.8, 86.2–100, 89.9–99.8, 94.2–99.3 and 83.8–99.8% in 2006, 2007, 2009, 2010, 2013 and 2014, respectively. Moreover, phylogenetic analysis demonstrated that the VP1 coding gene of the FMDV serotype O was clustered into two distinct viral topotypes, the SEA (lineage Mya-98) and ME-SA (lineage PanAsia) (Fig. 2). Most of the strains were clustered into the SEA topotype, while only the strains isolated between 2013 and 2014 were clustered into the ME-SA topotype (Fig. 2). The study strains shared nucleotide identities at a level of 89.0–92.6 and 84.1–86.6% compared to the O/MYA/2/2000/SEA (SEA) and O/A/CHA/58 (ME-SA) prototype strains, respectively [7, 22].

**Fig. 3:**
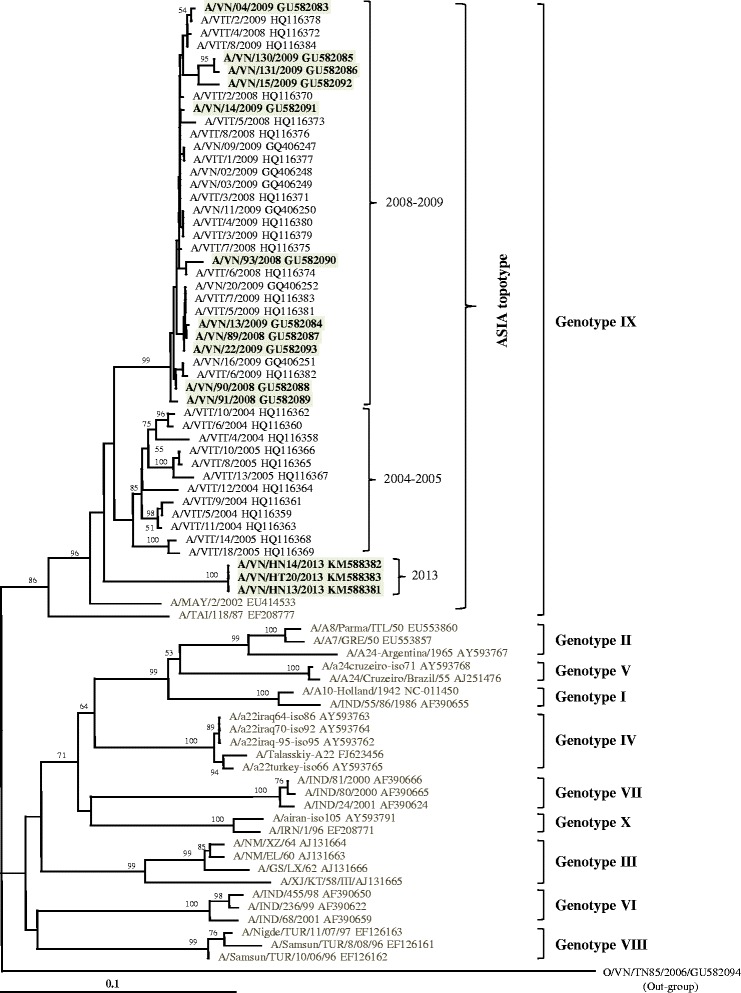
Phylogenetic tree based on the complete nucleotide sequence of the VP1 coding region of type A FMDVs showing relationships between the study strains and other type A representatives worldwide. The Vietnamese strains are marked in *bold*. *Numbers* at nodes indicate the level of bootstrap support based on the neighbor-joining analysis of 1000 re-sampled datasets. Only values above 50% are given. A *bar* represents 0.1 substitutions per nucleotide position

For FMDV serotype A, the VP1 nucleotide sequence identity among the study strains was 89.3–100%. Phylogenetic analysis indicated that the VP1 coding gene of the 14 FMDV serotype A strains was classified within the genotype IX (topotype Asia), together with other Vietnamese strains isolated during 2004–2009 (Fig. 3). The study strains showed nucleotide identity at a level of 87.9–91.1% compared to the A/TAI/118/87 prototype strain. Moreover, these FMDV serotype A showed genetic diversity among the strains circulating in Vietnam in the 2004–2005, 2008–2009, and 2013 seasons by grouping into three distinct sub-clusters (Fig. 3).

### Comparison of VP1 amino acid sequences

**Fig. 4:**
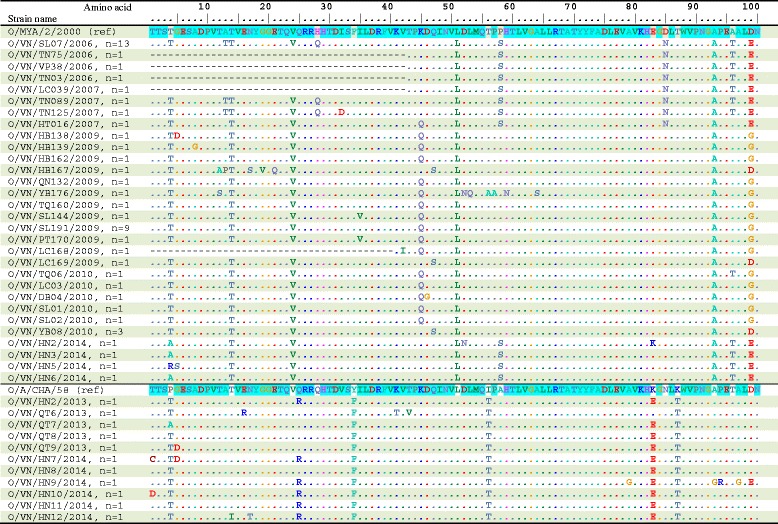
Deduced amino acid sequences of the VP1 proteins (aa 1-100) of the type O FMDVs in this study. Only sequences different from the consensus are shown. Strains with similar profiles of the antigenic site are grouped together. Similar amino acid sequences of the two reference trains, the O/MYA/2/2000 strain (for the SEA group) and the O/A/CHA/58 strain (for the ME-SA group), are shadowed. The VP1 antigenic sites are at amino acid position 43, 44, 45, 144, 148, 149, 154, and 208. The “n” represents the strains with similar profiles to the antigenic site

**Fig. 5:**
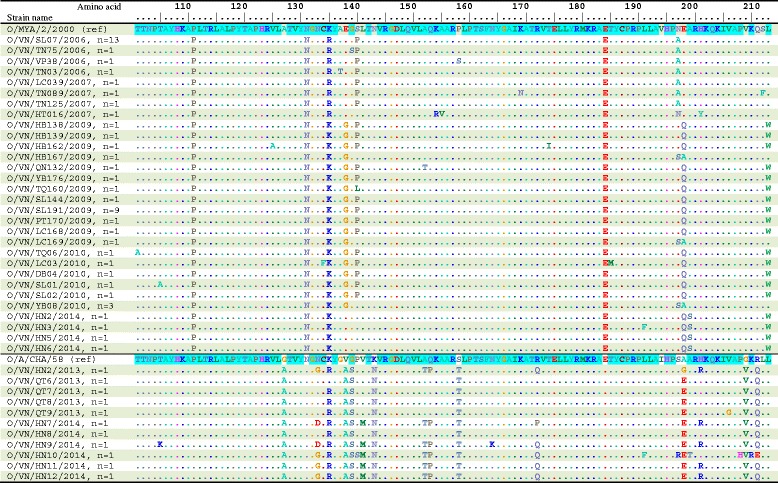
Deduced amino acid sequences of the VP1 proteins (aa 101-213) of the type O FMDVs in this study. Only sequences different from the consensus are shown. Strains with similar profiles of the antigenic site are grouped together. Similar amino acid sequences of the two reference trains, the O/MYA/2/2000 strain (for the SEA group) and the O/A/CHA/58 strain (for the ME-SA group), are shadowed. The VP1 antigenic sites are at amino acid position 43, 44, 45, 144, 148, 149, 154, and 208. The “n” represents the strains with similar profiles to the antigenic site

The deduced amino acid sequences obtained from the VP1 gene segments were aligned and compared to investigate the consequences of the observed genetic characterization of FMDV serotype O and A in Vietnam during 2006–2014. For the FMDV serotype O, the O/MYA/2/2000/SEA and O/A/CHA/58 prototype strains were used as references. Among the SEA topotype (lineage Mya-98), amino acid sequence identities significantly showed genetic variation of 90.1–100% within the Vietnamese strains and 89.6–93.4% to the O/MYA/2/2000 prototype strain. Compared to the O/MYA/2/2000/SEA prototype strain, seven amino acid substitutions were detected at positions 14, 24, 51, 93, 111, 131, and 184 (Figs. 4 and 5). Among the ME-SA topotype (lineage PanAsia), the study strains shared the amino acid identity at a level of 92.9–100% and shared an identity with O/A/CHA/58 prototype strain at a level of 86.8–93.4%. Eight amino acid substitutions were detected at positions 34, 56, 87, 127, 138, 139, 143, and 158 (Figs. 4 and 5). Notably, the change of the two amino acids at positions 45^K-Q^ and 154^K-R^ occurring at residues among the SEA topotypes, as well as the change of two amino acids at positions 43^T-V^ and 208^P-H^ occurring at residues among the ME-SA topotypes (Figs. 4 and 5).

**Fig. 6:**
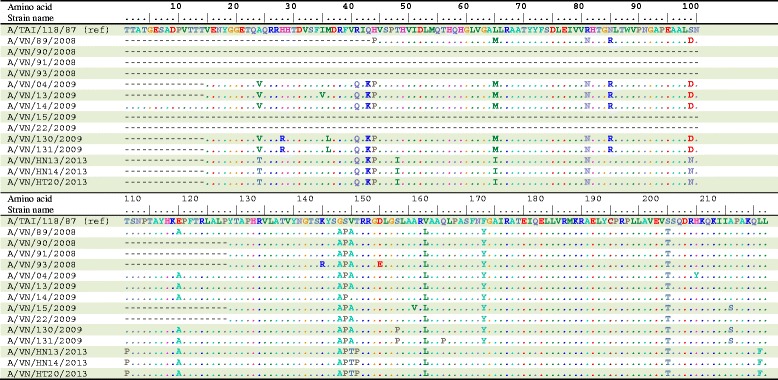
Deduced amino acid sequences of the VP1 proteins of the type A FMDVs in this study. Only sequences different from the consensus are shown. Strains with similar profiles of the antigenic site are grouped together. The VP1 antigenic sites are at amino acid position 43, 44, 45, 144, 148, 149, 154, and 208

For the FMDV serotype A, the A/TAI/118/87 prototype strain was used as a reference strain for genotype IX. Within the genotype IX (topotype Asia), the study strains shared 92.8–100% at the amino acid level and shared an identity with the A/TAI/118/87 prototype strain at level of 91.1–93.4%. Compared to the A/TAI/118/87 prototype strain, four amino acid substitutions were detected at positions 138, 139, 153, and 195 (Fig. 6). An amino acid changes in the antigenic sites noted at position 148^S-P^ of the A/VN/130/2009 and A/VN/131/2009 strains (Fig. 6).

## Discussion

The sequences of the VP1 coding gene are widely used to identify and characterize FMDV lineages and sub-lineages [23]. In addition, the VP1 capsid protein is the most helpful protein to investigate the relationship between different isolates of the FMDV because of its significance for viral attachment and entry, protective immunity, and serotype specificity [18, 19]. In this study, we used the VP1 coding region of the FMDV serotype O and type A isolated from the north and northern central regions of Vietnam during 2006–2014 for the determination of their phylogeny and genetic relationships with other available Vietnamese and global FMDV strains in the NCBI GeneBank database. Importantly, the 19 provinces enrolled in this study have historically been highly affected by FMDVs and share both a border and a trade of animals with the highly affected FMDV countries of China, Laos, Cambodia, and Thailand [9, 14, 16].

The FMDV serotype O is the most prevalent out of the seven serotypes that circulate in many parts of the world. The FMD outbreaks of type O viruses were first identified in 1987 from FMD outbreaks in Europe by analysis of its nucleotide sequence [24]. Based on the accumulation of VP1 genome sequences, 10 topotypes of FMDV serotype O were designated as the Euro-SA, ME-SA, SEA, CHY, WA, EA-1, EA-2, EA-3, ISA-1, and ISA-2. The topotypes ME-SA and SEA highly affect China, the Indian subcontinent (India, Pakistan, Bangladesh, Sri Lanka, Nepal, and Bhutan), and Southeast Asian countries (Myanmar, Thailand, Cambodia, Malaysia, Laos, and Vietnam). The Vietnamese FMDV serotype O fell within the ME-SA (lineage Mya-98), SEA (lineage PanAsia), and Cathay topotypes in which the isolates form distinct genetic sub-lineages and are distant from the prototype isolates. These findings highlight that these topotypes might have adapted in recent years to circulate in Vietnam.

The FMDV serotype A has been reported in all FMDV infected areas around the world. Based on the phylogeny analysis of the VP1 capsid genes, the global FMDV serotype A is divided into 10 major genotypes (designated as I to X) with over 15% nucleotide divergence [5, 6]. In Vietnam, these viruses were reported to be predominant during the outbreaks between 2008 and 2009 in the northern central regions of the country [16]. Genetic characterization of six serotype A strains isolated from the northern central regions of Vietnam revealed that the Vietnamese FMDV serotype A strains were all clustered into the genotype IX (topotype ASIA) and shared close relation to the recent FMDV serotype A strains isolated in Laos, Thailand, and Malaysia [12]. The FMDV serotype A strains in this study were also clustered into the genotype IX, together with the strains isolated between 2004 and 2009. Interestingly, the strains isolated in 2013 clustered into a single sub-cluster and showed distance from previous isolates. These results indicated the genetic variation of the FMDV serotype A strains and their persistent circulation in Vietnam, particularly in the north and northern central regions.

Available since the early 1900s, the FMDV vaccine continues to plays a significant role in the protection of animals against FMDV-related morbidity and mortality; however, the vaccinated individuals could only be protected for a specific serotype and/or subtype, and this protection is only valid for a short term [25]. The concurrent circulation of FMD outbreaks of the FMDV serotype O, A, and Asia 1 and its genetic variation may apply pressure for the selection of effective vaccine strains to prevent and control FMDV outbreaks in Vietnam [15]. These points also suggest that more FMDV surveillance studies will be necessary in order to evaluate the genetic relationship and efficacy of the current used vaccines against different FMDV serotypes circulating in Vietnam.

## Conclusions

This study provides valuable information on the genetic variation among the FMDVs serotype O and A circulating in Vietnam during 2006–2014, and likely indicates transmission between neighboring countries in Southeast Asia, such as Myanmar, Thailand, Cambodia, Malaysia, and Laos.

## Additional file

Additional file 1: Vietnamese serotypes O and FMDVs used in this study. (DOCX 33 kb)

## Abbreviations

CHY: Cathay
EA-1: East Africa 1
EA-2: East Africa 2
EA-3: East Africa 3
EA-4: East Africa 4
ELISA: Enzyme-linked immunosorbent assay
Euro-SA: Europe-South America
FMDV: Foot-and-mouth disease virus
ISA-1: Indonesia-1
ISA-2: Indonesia-2
ME-SA: Middle East-South Asia
RT-PCR: Reverse transcription polymerase chain reaction
SAT: South African Territories
SEA: Southeast Asia
WA: West Africa
