# Supplementary material for: Evolutionary phylodynamics of foot-and-mouth disease virus serotypes O and A circulating in Vietnam
Source: BMC Vet Res. 2016 Nov 29;12:269. doi: 10.1186/s12917-016-0896-0 (PMC5126991; doi:10.1186/s12917-016-0896-0)
Supplement: Additional file 1: — Vietnamese serotypes O and FMDVs used in this study. (DOCX 33 kb) [file 12917_2016_896_MOESM1_ESM.docx]

**Additional file 1**. Vietnamese serotypes O and A FMDVs used in this study.

| **Virusdesignation** | **Date collected** | **Geographical origin** | **Species** | **Type** | **Topotype** | **Accession number** |
| --- | --- | --- | --- | --- | --- | --- |
| This study | | | | | | |
| O/VN/VP38/2006 | 06/2006 | Vinh Phuc | Buffalo | O | SEA | GU582096 |
| O/VN/TN75/2006 | 07/2006 | Thai Nguyen | Cattle | O | SEA | GU582099 |
| O/VN/TN85/2006 | 10/2006 | Thai Nguyen | Buffalo | O | SEA | GU582094 |
| O/VN/TN03/2006 | 10/2006 | Thai Nguyen | Cattle | O | SEA | GU582107 |
| O/VN/HN18/2006 | 08/2006 | Ha Noi | Cattle | O | SEA | GU582095 |
| O/VN/HN83/2006 | 10/2006 | Ha Noi | Pig | O | SEA | GU582103 |
| O/VN/LS91/2006 | 10/2006 | Lang Son | Cattle | O | SEA | GU582102 |
| O/VN/LS08/2006 | 10/2006 | Lang Son | Pig | O | SEA | GU582105 |
| O/VN/SL601/2006 | 10/2006 | Son La | Pig | O | SEA | GU582101 |
| O/VN/SL6601/2006 | 10/2006 | Son La | Cattle | O | SEA | GU582106 |
| O/VN/SL607/2006 | 10/2006 | Son La | Cattle | O | SEA | GU582104 |
| O/VN/SL07/2006 | 10/2006 | Son La | Cattle | O | SEA | GU582097 |
| O/VN/SL6622/2006 | 10/2006 | Son La | Buffalo | O | SEA | GU582108 |
| O/VN/SL12/2006 | 10/2006 | Son La | Cattle | O | SEA | GU582100 |
| O/VN/SL622/2006 | 10/2006 | Son La | Buffalo | O | SEA | GU582098 |
| O/VN/SL06622/2006 | 10/2006 | Son La | Cattle | O | SEA | GU582109 |
| O/VN/LC039/2007 | 01/2007 | Lai Chau | Buffalo | O | SEA | GU582110 |
| O/VN/TN089/2007 | 02/2007 | Thai Nguyen | Cattle | O | SEA | GU582112 |
| O/VN/TN125/2007 | 04/2007 | Thai Nguyen | Cattle | O | SEA | GU582111 |
| O/VN/HT016/2007 | 12/2007 | Ha Tinh | Cattle | O | SEA | GU582113 |
| O/VN/YB106/2009 | 9/2009 | Yen Bai | Buffalo | O | SEA | GU582117 |
| O/VN/YB176/2009 | 11/2009 | Yen Bai | Buffalo | O | SEA | HM055503 |
| O/VN/SL109/2009 | 09/2009 | Son La | Buffalo | O | SEA | GU582118 |
| O/VN/SL144/2009 | 10/2009 | Son La | Buffalo | O | SEA | HM055495 |
| O/VN/SL145/2009 | 10/2009 | Son La | Buffalo | O | SEA | HM055496 |
| O/VN/SL186/2009 | 12/2009 | Son La | Buffalo | O | SEA | HM055505 |
| O/VN/SL187/2009 | 12/2009 | Son La | Pig | O | SEA | HM055506 |
| O/VN/SL191/2009 | 12/2009 | Son La | Buffalo | O | SEA | HM055507 |
| O/VN/SL192/2009 | 12/2009 | Son La | Buffalo | O | SEA | HM055508 |
| O/VN/HG128/2009 | 09/2009 | Ha Giang | Cattle | O | SEA | GU582119 |
| O/VN/TQ160/2009 | 10/2009 | Tuyen Quang | Buffalo | O | SEA | HM055497 |
| O/VN/NA79/2009 | 07/2009 | Nghe An | Cattle | O | SEA | GU582114 |
| O/VN/QN132/2009 | 01/2009 | Quang Ninh | Buffalo | O | SEA | GU582120 |
| O/VN/QN133/2009 | 01/2009 | Quang Ninh | Buffalo | O | SEA | GU582121 |
| O/VN/HB138/2009 | 01/2009 | Hoa Binh | Buffalo | O | SEA | GU582122 |
| O/VN/HB139/2009 | 10/2009 | Hoa Binh | Buffalo | O | SEA | HM055494 |
| O/VN/HB162/2009 | 10/2009 | Hoa Binh | Buffalo | O | SEA | HM055498 |
| O/VN/HB166/2009 | 10/2009 | Hoa Binh | Cattle | O | SEA | HM055499 |
| O/VN/HB167/2009 | 10/2009 | Hoa Binh | Cattle | O | SEA | HM055509 |
| O/VN/LS185/2009 | 12/2009 | Lang Son | Buffalo | O | SEA | HM055504 |
| O/VN/LC168/2009 | 11/2009 | Lao Cai | Cattle | O | SEA | HM055500 |
| O/VN/PT170/2009 | 11/2009 | Phu Tho | Cattle | O | SEA | HM055501 |
| O/VN/PT171/2009 | 11/2009 | Phu Tho | Cattle | O | SEA | HM055502 |
| O/VN/SL01/2010 | 02/2010 | Son La | Buffalo | O | SEA | HQ260713 |
| O/VN/SL02/2010 | 02/2010 | Son La | Buffalo | O | SEA | HQ260714 |
| O/VN/LC03/2010 | 02/2010 | Lao Cai | Buffalo | O | SEA | HQ260715 |
| O/VN/DB04/2010 | 02/2010 | Dien Bien | Cattle | O | SEA | HQ260716 |
| O/VN/TQ06/2010 | 02/2010 | Tuyen Quang | Buffalo | O | SEA | HQ260717 |
| O/VN/YB08/2010 | 02/2010 | Yen Bai | Cattle | O | SEA | HQ260718 |
| O/VN/YB09/2010 | 02/2010 | Yen Bai | Cattle | O | SEA | HQ260719 |
| O/VN/YB10/2010 | 02/2010 | Yen Bai | Buffalo | O | SEA | HQ260720 |
| O/VN/HN2/2013 | 04/2013 | Ha Noi | Porcine | O | ME-SA | KM588384 |
| O/VN/QT6/2013 | 10/2013 | Quang Tri | Bovine | O | ME-SA | KM588385 |
| O/VN/QT7/2013 | 10/2013 | Quang Tri | Bovine | O | ME-SA | KM588386 |
| O/VN/QT8/2013 | 10/2013 | Quang Tri | Bovine | O | ME-SA | KM588387 |
| O/VN/QT9/2013 | 10/2013 | Quang Tri | Bovine | O | ME-SA | KM588388 |
| O/VN/HN2/2014 | 03/2014 | Ha Nam | Porcine | O | SEA | KM588389 |
| O/VN/HN3/2014 | 03/2014 | Ha Nam | Porcine | O | SEA | KM588390 |
| O/VN/HN5/2014 | 03/2014 | Ha Nam | Porcine | O | SEA | KM588391 |
| O/VN/HN6/2014 | 03/2014 | Ha Nam | Bovine | O | SEA | KM588392 |
| O/VN/HN7/2014 | 03/2014 | Ha Noi | Porcine | O | ME-SA | KM588393 |
| O/VN/HN8/2014 | 03/2014 | Ha Noi | Porcine | O | ME-SA | KM588394 |
| O/VN/HN9/2014 | 03/2014 | Ha Noi | Porcine | O | ME-SA | KM588395 |
| O/VN/HN10/2014 | 06/2014 | Ha Noi | Porcine | O | ME-SA | KM588396 |
| O/VN/HN11/2014 | 06/2014 | Ha Noi | Porcine | O | ME-SA | KM588397 |
| O/VN/HN12/2014 | 06/2014 | Ha Noi | Porcine | O | ME-SA | KM588398 |
| A/VN/89/2008 | 12/2008 | Nghe An | Buffalo | A | Genotype IX | GU582087 |
| A/VN/90/2008 | 12/2008 | Nghe An | Buffalo | A | Genotype IX | GU582088 |
| A/VN/91/2008 | 12/2008 | Nghe An | Cattle | A | Genotype IX | GU582089 |
| A/VN/93/2008 | 12/2008 | Nghe An | Buffalo | A | Genotype IX | GU582090 |
| A/VN/04/2009 | 02/2009 | Hoa Binh | Buffalo | A | Genotype IX | GU582083 |
| A/VN/13/2009 | 02/2009 | Bac Can | Cattle | A | Genotype IX | GU582084 |
| A/VN/14/2009 | 02/2009 | Bac Can | Buffalo | A | Genotype IX | GU582091 |
| A/VN/15 2009 | 02/2009 | Phu Tho | Buffalo | A | Genotype IX | GU582092 |
| A/VN/130/2009 | 09/2009 | Ha Giang | Cattle | A | Genotype IX | GU582085 |
| A/VN/131/2009 | 09/2009 | Ha Giang | Cattle | A | Genotype IX | GU582086 |
| A/VN/22/2009 | 02/2009 | Quang Tri | Buffalo | A | Genotype IX | GU582093 |
| A/VN/HT20/2013 | 10/2013 | Ha Tinh | Bovine | A | Genotype IX | KM588383 |
| A/VN/HN13/2013 | 12/2013 | Ha Noi | Bovine | A | Genotype IX | KM588381 |
| A/VN/HN14/2013 | 12/2013 | Ha Noi | Bovine | A | Genotype IX | KM588382 |
| Available in NCBI GeneBank database | | | | | | |
| O/VIT/7/97 | 1997 | Vietnam | - | O | SEA | AJ296328 |
| O/VIT/4/2005 | 2005 | Vietnam | Cattle | O | SEA | HQ116278 |
| O/VIT/6/2005 | 3/2005 | Vietnam | Cattle | O | SEA | HQ116279 |
| O/VN/SL22/2006 | 10/2006 | Son La | Cattle | O | SEA | GU125647 |
| O/VN/SL01/2006 | 10/2006 | Son La | Buffalo | O | SEA | GU125648 |
| O/VN/GL13/2006 | 4/2006 | Vietnam | Cattle | O | SEA | GU125650 |
| O/VIT/4/2006 | 10/2006 | Vietnam | Pig | O | SEA | HQ116287 |
| O/VIT/5/2006 | 2/2006 | Vietnam | Cattle | O | SEA | HQ116288 |
| O/VIT/6/2006 | 3/2006 | Vietnam | Pig | O | SEA | HQ116289 |
| O/VIT/7/2006 | 3/2006 | Vietnam | Cattle | O | SEA | HQ116290 |
| SonLa-2/VIT/06 | 10/2006 | Son La | Cattle | O | SEA | GQ855799 |
| SonLa-3/VIT/06 | 10/2006 | Son La | Buffalo | O | SEA | GQ855800 |
| Hanoi/VIT/06 | 10/2006 | Ha Noi | Pig | O | SEA | GQ855801 |
| SonLa-1/VIT/06 | 10/2006 | Son La | Cattle | O | SEA | GQ855802 |
| NgheAn/VIT/07 | 12/2007 | Nghe An | Cattle | O | SEA | GQ855804 |
| HaTinh/VIT/07 | 12/2007 | Ha Tinh | Cattle | O | SEA | GQ855805 |
| O/VN/LC169/2009 | 11/2009 | Lao Cai | - | O | SEA | HM055510 |
| O/VIT/17/99 | 1999 | Vietnam | - | O | ME-SA | AJ318858 |
| O/VIT/6/2002 | 2002 | Vietnam | - | O | ME-SA | DQ165020 |
| O/VIT/7/2002 | 2002 | Vietnam | Cattle | O | ME-SA | HQ116273 |
| O/VIT/8/2002 | 2002 | Vietnam | - | O | ME-SA | DQ165021 |
| O/VIT/9/2002 | 2002 | Vietnam | - | O | ME-SA | DQ165022 |
| O/VIT/10/2002 | 2002 | Vietnam | - | O | ME-SA | DQ165023 |
| O/VIT/12/2002 | 2002 | Vietnam | - | O | ME-SA | DQ165024 |
| O/VIT/14/2002 | 2002 | Vietnam | Porcine | O | ME-SA | DQ165026 |
| O/VIT/16/2002 | 2002 | Vietnam | Porcine | O | ME-SA | DQ165027 |
| O/VIT/19/2002 | 2002 | Vietnam | Bovine | O | ME-SA | DQ165028 |
| O/VIT/20/2002 | 2002 | Vietnam | Bovine | O | ME-SA | DQ165029 |
| O/VIT/1/2003 | 2003 | Vietnam | Porcine | O | ME-SA | DQ165030 |
| O/VIT/2/2003 | 2003 | Vietnam | Porcine | O | ME-SA | DQ165031 |
| O/VIT/1/2004 | 2004 | Vietnam | Bovine | O | ME-SA | DQ165032 |
| O/VIT/7/2004 | 2004 | Vietnam | Cattle | O | ME-SA | HQ116274 |
| O/VIT/8/2004 | 2004 | Vietnam | Cattle | O | ME-SA | HQ116275 |
| O/VIT/3/2005 | 2005 | Vietnam | Cattle | O | ME-SA | HQ116277 |
| O/VIT/7/2005 | 2005 | Vietnam | Cattle | O | ME-SA | HQ116280 |
| O/VIT/17/2005 | 2005 | Vietnam | Cattle | O | ME-SA | HQ116283 |
| KhanhHoa/VIT/06 | 10/2006 | Khanh Hoa | - | O | ME-SA | GQ855798 |
| O/VIT/3/97 | 1997 | Vietnam | Porcine | O | Cathay | AJ294930 |
| O/VIT/2/99 | 1999 | Vietnam | - | O | Cathay | AJ318857 |
| O/VIT/13/2002 | 2002 | Vietnam | - | O | Cathay | DQ165025 |
| O/VIT/3/2004 | 3/2004 | Vietnam | Porcine | O | Cathay | DQ165034 |
| O/VIT/2/2004 | 2004 | Vietnam | Porcine | O | Cathay | DQ165033 |
| O/VIT/1/2005 | 2005 | Vietnam | Cattle | O | Cathay | HQ116276 |
| O/VIT/9/2005 | 2005 | Vietnam | Porcine | O | Cathay | HQ116281 |
| O/VIT/11/2005 | 6/2005 | Vietnam | Porcine | O | Cathay | HQ116282 |
| O/VIT/1/2006 | 2006 | Vietnam | Porcine | O | Cathay | HQ116284 |
| O/VIT/2/2006 | 2006 | Vietnam | Porcine | O | Cathay | HQ116285 |
| O/VIT/3/2006 | 2006 | Vietnam | Porcine | O | Cathay | HQ116286 |
| ThuaThienHue/VIT/07 | 7/2007 | Hue | Porcine | O | Cathay | GQ855803 |
| O/VIT/1/2008 | 2008 | Vietnam | Porcine | O | Cathay | HQ116291 |
| A/VIT/4/2004 | 2004 | Vietnam | Cattle | A | Genotype IX | HQ116358 |
| A/VIT/5/2004 | 2004 | Vietnam | Cattle | A | Genotype IX | HQ116359 |
| A/VIT/6/2004 | 2004 | Vietnam | Cattle | A | Genotype IX | HQ116360 |
| A/VIT/9/2004 | 2004 | Vietnam | Cattle | A | Genotype IX | HQ116361 |
| A/VIT/10/2004 | 2004 | Vietnam | Cattle | A | Genotype IX | HQ116362 |
| A/VIT/11/2004 | 2004 | Vietnam | Cattle | A | Genotype IX | HQ116363 |
| A/VIT/12/2004 | 2004 | Vietnam | Cattle | A | Genotype IX | HQ116364 |
| A/VIT/8/2005 | 2005 | Vietnam | Cattle | A | Genotype IX | HQ116365 |
| A/VIT/10/2005 | 2005 | Vietnam | Cattle | A | Genotype IX | HQ116366 |
| A/VIT/13/2005 | 2005 | Vietnam | Cattle | A | Genotype IX | HQ116367 |
| A/VIT/14/2005 | 2005 | Vietnam | Cattle | A | Genotype IX | HQ116368 |
| A/VIT/18/2005 | 2005 | Vietnam | Cattle | A | Genotype IX | HQ116369 |
| A/VIT/2/2008 | 2008 | Vietnam | Cattle | A | Genotype IX | HQ116370 |
| A/VIT/3/2008 | 2008 | Vietnam | Cattle | A | Genotype IX | HQ116371 |
| A/VIT/4/2008 | 2008 | Vietnam | Buffalo | A | Genotype IX | HQ116372 |
| A/VIT/5/2008 | 2008 | Vietnam | Cattle | A | Genotype IX | HQ116373 |
| A/VIT/6/2008 | 2008 | Vietnam | Cattle | A | Genotype IX | HQ116374 |
| A/VIT/7/2008 | 2008 | Vietnam | Buffalo | A | Genotype IX | HQ116375 |
| A/VIT/8/2008 | 2008 | Vietnam | Cattle | A | Genotype IX | HQ116376 |
| A/VIT/1/2009 | 2009 | Vietnam | Cattle | A | Genotype IX | HQ116377 |
| A/VIT/2/2009 | 2009 | Vietnam | Buffalo | A | Genotype IX | HQ116378 |
| A/VIT/3/2009 | 2009 | Vietnam | Buffalo | A | Genotype IX | HQ116379 |
| A/VIT/4/2009 | 2009 | Vietnam | Cattle | A | Genotype IX | HQ116380 |
| A/VIT/5/2009 | 2009 | Vietnam | Buffalo | A | Genotype IX | HQ116381 |
| A/VIT/6/2009 | 2009 | Vietnam | Pig | A | Genotype IX | HQ116382 |
| A/VIT/7/2009 | 2009 | Vietnam | Buffalo | A | Genotype IX | HQ116383 |
| A/VIT/8/2009 | 2009 | Vietnam | Buffalo | A | Genotype IX | HQ116384 |
| A/VN/02/2009 | 2009 | Vietnam | Cattle | A | Genotype IX | GQ406248 |
| A/VN/03/2009 | 2009 | Vietnam | Cattle | A | Genotype IX | GQ406249 |
| A/VN/09/2009 | 2009 | Vietnam | Cattle | A | Genotype IX | GQ406247 |
| A/VN/11/2009 | 2009 | Vietnam | Cattle | A | Genotype IX | GQ406250 |
| A/VN/16/2009 | 2009 | Vietnam | Pig | A | Genotype IX | GQ406251 |
| A/VN/20/2009 | 2009 | Vietnam | Buffalo | A | Genotype IX | GQ406252 |
